# Supplementary material for: Re-engagement and retention in HIV care after preventive default tracking in a cohort of HIV-infected patients in rural Malawi: A mixed-methods study
Source: PLOS Glob Public Health. 2024 Feb 21;4(2):e0002437. doi: 10.1371/journal.pgph.0002437 (PMC10880992; doi:10.1371/journal.pgph.0002437)
Supplement: S1 Checklist — (DOCX) [file pgph.0002437.s001.docx]

STROBE Statement—checklist of items that should be included in reports of observational studies

|  | Item No. | Recommendation | Page  No. | Relevant text from manuscript |
| --- | --- | --- | --- | --- |
| **Title and abstract** | 1 | (*a*) Indicate the study’s design with a commonly used term in the title or the abstract | 1 | **Re-engagement and retention in HIV care after preventive default tracking in a cohort of HIV-infected patients in rural Malawi: A mixed-methods study** |
|  |  | (*b*) Provide in the abstract an informative and balanced summary of what was done and what was found | 2 | We utilized a mixed-methods study to evaluate the impact of the TRACE program on patient’s re-engagement and retention in care (RiC). We found that after tracking, 982 (96%) of patients with a 6-week missed appointment returned to care. After returning to care, 906 (88%), 864 (84%), and 839 (82%) were retained in care respectively at 6,12, and 24 months. |
| Introduction | | | |  |
| Background/rationale | 2 | Explain the scientific background and rationale for the investigation being reported | 3 | Human immunodeficiency virus (HIV) is still a significant challenge globally, with 38.6 million people living with HIV (PLHIV) at the end of 2021(1). In Sub-Saharan Africa, 56% of HIV program attrition is attributed to LTFU (12) and is prevalent among men, women, and children, as well as other specific groups living with HIV (13–15). In Western Kenya, the incidence of LTFU was 28.1% and 23.8 per 100 person-year in men and women between 2001 and 2007(13). Leshargie and al, in data published between 2005 and 2020 that the trend of LTFU in adolescents living with HIV and initiated into treatment in Sub-Saharan Africa was increasing over time and older adolescents (15-19 years old) 43% were more likely to be LTFU than the younger ones (10-14 years old)(16). Loss to follow-up has been reported to double the mortality risk for HIV-infected children who started treatment in South Africa (17). Furthermore, even if patients return to care after being LTFU, they have been reported to be more likely to die than patients who attended the clinic regularly (18). With irregular treatment and follow-up, patients who have left care have been reported to be a significant driver for new cases of HIV infections (19). As patients engage and disengage throughout the continuum of care, LTFU constitutes a real threat to test-and-treat success in Malawi. To combat this threat, the World Health Organization (WHO) has recommended that HIV care programs implement clinical and community-based interventions to support ART adherence and patient RiC (28,29). Intervention examples for LTFU patients include phone calls, text messages, peer counsellors, patient navigators, or lay counsellors to support adherence to ART (30–36). Recognizing the detrimental effect of LTFU on HIV programs, we created a preventive default tracking strategy named the Tracking Retention And Care Enrollment (TRACE) program. This program was implemented in 2016 to prevent patients from becoming LTFU in the rural Neno District of southeastern Malawi. The TRACE program is preventive and focuses on the linkage between the health facility and the patient by providing patient-centered support. By aiming to prevent LTFU, TRACE improves retention, hopefully improving viral load suppression and overall patient outcomes. That said, this program has not been formally evaluated. Further knowledge and outcome evidence of the TRACE program will serve to inform design and implementation of this program and other community interventions that aim to prevent LTFU and increase patient re-engagement. |
| Objectives | 3 | State specific objectives, including any prespecified hypotheses | 4 | . Therefore, we aimed to assess patient re-engagement, RiC, and patient characteristics after being tracked by the TRACE program. We also wished to assess program acceptability from the perspectives of the beneficiaries in a mixed-methods evaluation. |
| Methods | | | |  |
| Study design | 4 | Present key elements of study design early in the paper | 1, 8, 9, 10, 11 | We utilized a mixed-methods study to evaluate the impact of the TRACE program on patient’s re-engagement and retention in care (RiC). In the quantitative arm, we utilized secondary data of HIV-infected patients in the TRACE program from January 2018 to June 2019 and analyzed patients ‘outcomes at 6, 12, and 24 months post-tracking. In the qualitative arm, we analyzed primary data from 25 semi-structured interviews. |
| Setting | 5 | Describe the setting, locations, and relevant dates, including periods of recruitment, exposure, follow-up, and data collection | 9,10,11 | De-identified data was collected using TRACE outcome monthly EMR reports and ART registers from EMR system at IC3 Clinics Neno Malawi. All 1100 patients tracked between January 2018 to June 2019 were included. From the pool of participants, 26 were invited but 25 were interviewed instead, as one had withdrawn from the study after being contacted. Recruitment and interviews were conducted between August to December 2021. |
| Participants | 6 | (*a*) *Cohort study*—Give the eligibility criteria, and the sources and methods of selection of participants. Describe methods of follow-up  *Case-control study*—Give the eligibility criteria, and the sources and methods of case ascertainment and control selection. Give the rationale for the choice of cases and controls  *Cross-sectional study*—Give the eligibility criteria, and the sources and methods of selection of participants | 9,10,11 | **For the quantitative arm:** All 1100 patients that were tracked between January 2018 to June 2019 were included in the analysis. Data source were TRACE outcome monthly EMR reports and ART registers from EMR system at IC3 Clinics. **For the Qualitative arm**: Only patients 18 years of age or older, who could give consent, were included in this study. Twenty-five in-depth semi-structured interviews were conducted with ART active patients purposefully selected from the list of patients tracked preventively and successfully between January 2018 and June 2019. |
|  |  | (*b*) *Cohort study*—For matched studies, give matching criteria and number of exposed and unexposed  *Case-control study*—For matched studies, give matching criteria and the number of controls per case |  |  |
| Variables | 7 | Clearly define all outcomes, exposures, predictors, potential confounders, and effect modifiers. Give diagnostic criteria, if applicable |  | **The primary study outcome** was retention after returning to care. Retention was measured from the day of re-engagement (i.e., the origin). A patient was to be considered retained at any given time if she/he at the time of analysis (6, 12, 24 months) was alive and enrolled in ART care or transferred to another HIV program after re-engagement in care. A patient was considered *not* retained if she/he defaulted, stopped ART, or died after re-engagement in care and did not reach the time of analysis (6, 12, 24 months). Return to care was also reported in this study and defined as any visit after the tracking visit within the study analysis of December 2021. |
| Data sources/ measurement | 8* | For each variable of interest, give sources of data and details of methods of assessment (measurement). Describe comparability of assessment methods if there is more than one group | *10* | When estimating retention (to compare the proportion retained after successful tracing), we used generalized estimating equations with a log link and binomial distribution to estimate the risk ratio at 95% confidence intervals. We used an exchangeable correlation matrix and a robust variance estimator to account for correlation. Multivariate logistic regression was also used to identify patient characteristics independently associated with the outcome of return to care after TRACE in the study population. No comparison group was included in the analysis. |
| Bias | 9 | Describe any efforts to address potential sources of bias | 33 | The sample used for the quantitative analysis was small, and this analysis was conducted in only one district.  However, the mixed-methods approach provides a comprehensive understanding of patient re-engagement and allows us to explore long-term RiC post-tracking, thus adding to and supporting the knowledge we already have regarding retention and re-engagement in care |
| Study size | 10 | Explain how the study size was arrived at |  | Since we were studying a finite population therefore all eligible patients were included in the study. |

Continued on next page

| Quantitative variables | 11 | Explain how quantitative variables were handled in the analyses. If applicable, describe which groupings were chosen and why | 8,10 | | The quantitative outcomes were 1) Re-engagement or return to care of patients out of care beyond 6 weeks of a missed visit 2) RiC at 6,12- and 24-months post tracking and 3) descriptive characteristics of these patients as factors associated with their retention in care post tracking. Variables reported were age, gender, year of ART initiation, BMI, CD4 at initiation, the reason for ART start, ART education, TB status at registration, and ART initiating facility. A chi-square test for continuous data was computed for validity (Table 1). Results for continuous variables, including age and year on ART, were presented by the median and interquartile range (IQR). Meanwhile, categorical variables such as age group, sex, BMI, ART start after test-and-treat era, CD4 at ART initiation, clinical staging at ART initiation, ART education session done (or not), and TB status at registration were presented as a proportion of the variables of the 1100 patients listed. The primary analytic approach was the intention to treat, where participants were analyzed in the category to which they were assigned, regardless of whether or how often they were seen by CHWs or TRACE field workers. | |
| --- | --- | --- | --- | --- | --- | --- |
| Statistical methods | 12 | (*a*) Describe all statistical methods, including those used to control for confounding | |  | |  |
|  |  | (*b*) Describe any methods used to examine subgroups and interactions | |  | | When estimating retention (to compare the proportion retained after successful tracing), we used generalized estimating equations with a log link and binomial distribution to estimate the risk ratio at 95% confidence intervals. We used an exchangeable correlation matrix and a robust variance estimator to account for correlation. Multivariate logistic regression was also used to identify patient characteristics independently associated with the outcome of return to care after TRACE in the study population. |
|  |  | (*c*) Explain how missing data were addressed | | 12 | | We excluded participants from the primary outcomes as they had started and transferred out on a day of registration when such information was missing on EMR records |
|  |  | (*d*) *Cohort study*—If applicable, explain how loss to follow-up was addressed  *Case-control study*—If applicable, explain how matching of cases and controls was addressed  *Cross-sectional study*—If applicable, describe analytical methods taking account of sampling strategy | |  | |  |
|  |  | (*e*) Describe any sensitivity analyses | |  | |  |
| Results | | | | | | |
| Participants | 13* | (a) Report numbers of individuals at each stage of study—eg numbers potentially eligible, examined for eligibility, confirmed eligible, included in the study, completing follow-up, and analysed | | 12 | | A total of 1100 individuals had a TRACE visit, and of these, 1028 patients met the inclusion criteria of physical patient verification at a facility on master cards and registers. We excluded 72 participants from the primary outcomes as they had started and transferred out on a day of registration when such information was missing on EMR records. |
|  |  | (b) Give reasons for non-participation at each stage | |  | |  |
|  |  | (c) Consider use of a flow diagram | |  | |  |
| Descriptive data | 14* | (a) Give characteristics of study participants (eg demographic, clinical, social) and information on exposures and potential confounders | | 9 | | This evaluation study included all HIV-positive patients in the Neno District IC3 HIV cohort who were tracked between January 2018 and June 2019. They had been tracked because they had missed their appointments for a clinical review or for a drug refill for 6 weeks or more. |
|  |  | (b) Indicate number of participants with missing data for each variable of interest | | 12 | | 72 |
|  |  | (c) *Cohort study*—Summarise follow-up time (eg, average and total amount) | |  | |  |
| Outcome data | 15* | *Cohort study*—Report numbers of outcome events or summary measures over time | |  | |  |
|  |  | *Case-control study—*Report numbers in each exposure category, or summary measures of exposure | |  | | In keeping with the Neno District TRACE strategy, patient tracking for retention is initiated at 2, 4, and 6 weeks (of missing a scheduled appointment) rather than waiting the full eight weeks, defined by the national guidelines, to prevent LTFU (Figure 1). |
|  |  | *Cross-sectional study—*Report numbers of outcome events or summary measures | |  | |  |
| Main results | 16 | (*a*) Give unadjusted estimates and, if applicable, confounder-adjusted estimates and their precision (eg, 95% confidence interval). Make clear which confounders were adjusted for and why they were included | | 16 | | Retention in Care: We used logistic regression with univariate and multivariate analyses to examine potential demographic and clinical factors associated with re-engagement in care at 6,12,and 24 months post-TRACE visit (Table 4). The multivariate model was constructed by including variables with a p-value<0.2 in the univariate analysis and further adjusting by age and gender. |
|  |  | (*b*) Report category boundaries when continuous variables were categorized | |  | |  |
|  |  | (*c*) If relevant, consider translating estimates of relative risk into absolute risk for a meaningful time period | |  | |  |

Continued on next page

| Other analyses | 17 | Report other analyses done—eg analyses of subgroups and interactions, and sensitivity analyses |  | |  |
| --- | --- | --- | --- | --- | --- |
| Discussion | | | | | |
| Key results | 18 | Summarise key results with reference to study objectives | 29 | |  |
| Limitations | 19 | Discuss limitations of the study, taking into account sources of potential bias or imprecision. Discuss both direction and magnitude of any potential bias | 32,33 | |  |
| Interpretation | 20 | Give a cautious overall interpretation of results considering objectives, limitations, multiplicity of analyses, results from similar studies, and other relevant evidence | 35 | | This study demonstrates the effectiveness of the TRACE strategy, with most patients returning to care and with high retention rates, especially among men, older individuals, and those at a more advanced clinical stage at enrollment, and as a result of universal test-and-treat guidelines. |
| Generalisability | 21 | Discuss the generalisability (external validity) of the study results | 33 | | The sample used for the quantitative analysis was small, and this analysis was conducted in only one district |
| Other information | |  | | | |
| Funding | 22 | Give the source of funding and the role of the funders for the present study and, if applicable, for the original study on which the present article is based |  | This study was made possible by the support of Wagner Foundation. Dr. Rosenthal’s role was funded by the Israel Science Foundation - ISF (Grant 1073/18). | |

*Give information separately for cases and controls in case-control studies and, if applicable, for exposed and unexposed groups in cohort and cross-sectional studies.

**Note:** An Explanation and Elaboration article discusses each checklist item and gives methodological background and published examples of transparent reporting. The STROBE checklist is best used in conjunction with this article (freely available on the Web sites of PLoS Medicine at http://www.plosmedicine.org/, Annals of Internal Medicine at http://www.annals.org/, and Epidemiology at http://www.epidem.com/). Information on the STROBE Initiative is available at www.strobe-statement.org.
