# Supplementary material for: Re-engagement and retention in HIV care after preventive default tracking in a cohort of HIV-infected patients in rural Malawi: A mixed-methods study
Source: PLOS Glob Public Health. 2024 Feb 21;4(2):e0002437. doi: 10.1371/journal.pgph.0002437 (PMC10880992; doi:10.1371/journal.pgph.0002437)
Supplement: S1 Data — (DOCX) [file pgph.0002437.s002.docx]

| **Code** | **Description** | **Supporting Quotes** |
| --- | --- | --- |
| Population of clients | **Age:** 19 to 70 years  **Gender:** Men and women  **Months on ART:** Poor adherence within the first 6 months  **Reg #:** N/A  **Adherence:** Excellent after traced back |  |
|  |  |  |
|  |  |  |
|  |  |  |
|  |  |  |
|  |  | ·       *But Aah eeh, I was just doing that intentionally I forgot, chose not to follow the date not to go on the right days and missing dates, but after being visited to advise me I can see that things are going on well I am keeping the dates, I am not missing the taking medication and I am taking following the dates every morning am taking medication.* **Trace client in NN-participant 09** |
| Reasons for defaulting | Factors that led to default among ART clients | ·       ***For*** *them to come it’s because I did not take medication, I had an argument with mom so I decided not to take medication I just made a choice not to take the medication.* **Trace client in NN-participant 06** |
|  |  | ·       *The reason why I missed to collect medication, I collected that month and went to the village where my father died, and when I left, so when I was there the date reached but I did not have transport to come and collect medication, so when I came here my in-law told me that a health worker came looking for me, so when I came I visited the health worker and they told me they will visit me to have conversation with me.* **Trace client in NN-participant 08** |
|  |  | ·       *What happened was that at the hospital there was a misunderstanding because my file was missing and most of the times they were telling me to come back because I was late, I visited afternoon hours when they were knocking off, and most of the times I was coming back because of those reason.* **Trace client in NN-participant 20** |
|  |  | ·       *… I forgot because they were laughing at me at home, when I quarrel with them they were running at me calling me a sick people with the disease.* **Trace client in NN-participant 01. Mmmh, I really stopped taking medication, what made me to stop taking medication is that people were talking a lot to me at this house when I touch the utensil they were saying I am going to infect them so I made a choice to stop taking medication so that I should die and forget things and get sick.Trace client in NN-participant 18** |
|  |  | ·       *I was just confused, what was in my heart was I was hurt it was not concerning health but in our village here I lost my sister and I was left alone so It was a burden and I decided to leave the area to seek for medical help of spiritual world because I was attacked by evil spirit so when I went there I run out of bacterium.* **Trace client in NN-participant 02** |
|  |  | ·       …*because I left this place and most of my relatives stay in Blantyre and when I get sick they don’t allow me to be here*. **Trace client in NN-participant 24** |
|  |  | ·       *Aaah on that I have already said that I have a disease that is bothering me and receiving this medication and it is not helping me, that is why I decided to stop taking the medication, and for those that will get cured from the medication they should be them but for me they are not helping me.* **Trace client in NN-participant 20** |
|  |  | I can say that it was helpful for those that visited me, because I have been explaining my problem to them but they did not hear me out, my problem was not solved, the people I was explaining to I thought they would help me solve it, I was just explaining that they are giving me the medication but the problem I was going through are like this, but I was never helped. Yes, I have it till today, I went to the hospital, that, at the beginning of the problem they carried out some biopsy in xxxx the same disease and when they removed the tissue they wanted they took it to Queens and when it went to queens the results did not come till today, the results did not come, it is when I started losing my interest of going to the hospital, they removed some of my organs and they did not tell me the results, and the person that removed those organs maybe you know him, and I said that they are not helping me. Trace client in NN-participant 20 |
|  |  | ·      *Because I did not have food and I went to cultivate maize… It’s when I told the (wind) food is a problem, food is a problem at this house, it’s not that I chose intentionally to fail to collect medication.* **Trace client in NN-participant 20. we are poor for example I work up early for some piece work taking medication without having breakfast because of money problems, and the medication affects the heart we work while we are sad, we just do because we have nothing to do, for me I am complaining that if you have some work employ us maybe our lives will move forward. Trace client in NN-participant 13** |
|  |  | ·      *Aaah firstly ,I would say I had an emergency journey which made me to forget the date of going back to the hospital.I was in Mozambique for business and it took me time to come back.* **Trace client in NN-participant 25** |
|  |  | ·       *The problem is that he was not taking medication and the medication were just accumulating, so I was just feeling lazy to collect some medication for him, since he was refusing to take medication, when I forced him he was refusing to take medication there was no proper reason for him not to take, not to collect medication.* ***Trace client in NN-participant 11*** |
|  |  | ·       *Yes I missed, I was sick so I made a choice not to go when I get better I did not know that I can collect medication at the hospital while I am sick.* ***Trace client in NN-participant 12*** |
|  |  | ·       *…because I was obsessed with alcohol and with my wife we confused each other the way we divorced and she threw away my goods and medication and it happened that I had no medication and I did not have time to go to the hospital, so I was just staying and it’s when they visited me.* ***Trace client in NN-participant 13*** |
|  |  | ·       *I forgot my date because sometimes they were giving us more medication for several month then I forgot my date to go, that was the reason they visited me because I did not go on those dates.* ***Trace client in NN-participant 17*** |
| Perceptions of trace | How clients perceive the trace system |  |
|  |  | ·       *… they went around to see who are not going to the clinic and they follow them to know what the problem is and those* *who this is not working they follow them, to know what is happening to the point of the viral load…not clear.* ***Trace*** ***client in NN-participant 11*** |
|  |  | ·       *They came to see me, with the way they came to see me as a friend not trace team and it when they told me to go to the hospital.* ***Trace*** ***client in NN-participant 11*** |
|  |  | … we are patients and we are happy for your visit and the work you are doing and when you go to the office sit down and talk.. Trace client in NN-participant 13 |
|  |  | ·       *They wanted me to be a good person, I mean when they left their jobs and visited me they wanted me to be in good health, I should be like my friends*. ***Trace*** ***client in NN-participant 11*** |
|  |  | ·       my thoughts is that when I stay I appreciate them they showed me the right way, they me light, they took me from the darkness and showed me light, I appreciate much even when I stay I appreciate them those people should help others in the future, they wished my life and I want to them to wish other peoples life as well. ***Trace*** ***client in NN-participant 18*** |
|  |  | ·       …motivate them to their health bodies and go back to clinic, I can just say it is to motivate them that their health is important and they should take care of their body, the trace team motivates them. ***Trace* *client in NN-participant 11*** |
|  |  | ·       *The conversation was very fruitful, because when I came I started taking medication properly since I don’t want them to come and remind me, so I follow what they told me and the doctors because the doctors take us in classes when we visit the hospital after being taught they tell us not to stop taking medication , it like when we stop we make this people repeat the same job several times when I am going to the hospital properly and taking medication collection this people doesn’t have tough time with me, because they know that am following everything properly****. Trace client in NN-participant 12*** |
|  |  | ·       *Trace is a group of doctors that work with us patient who are discourage the walk around the house and it’s a good house, they remind us when we forget about taking medication and tell us to go, for me I know that they visit and encourage me when I am discouraged the come and find me and ask what has happened if I am sick, I don’t have a guardian.* ***Trace client in NN-participant 13*** |
|  |  | ·       *what I know is when people are not collecting medication and they follow them and when it happens that after following them they don’t go to the hospital they go visit them again, and they can sometimes leave and told the person that when he gets sick they will not provide any assistance maybe that is what I know, the main aim of the visit is to find out if our body are healthy, maybe we can be collecting medication without taking them, and they also see that the days are accumulating at the hospital, and they visit us to see if we are taking medication properly, to prevent sickness that comes in the future, and it can happen that they have lost their person so they encourage the person that everyone should be taking medication at the right time and live a healthy life.* ***Trace client in NN-participant 17*** |
|  |  | ·       *Mmmh****,*** *I really stopped taking medication, what made me to stop taking medication is that people were talking a lot to me at this house when I touch the utensil they were saying I am going to infect them so I made a choice to stop taking medication so that I should die and forget things and get sick that was when I saw friends that loved me followed me and made me start taking medication, right now I see that when people talk a lot about I should only care about my life.* ***Trace client in NN-participant 18*** |
| Benefit of trace to clients | What clients perceive as trace benefits or advantages to them |  |
|  |  | ·       *It motivates us to see the date when to collect medication at the hospital at the right time, because of trace we are surprised and reminded to collect medication when they visit me today, I try my best not to be visited again to remind me, so it helps, helps to know time to collect medication is here.* ***Trace*** ***client in NN-participant 11*** |
|  |  | ·       *intentionally I forgot, chose not to follow the date not to go on the right days and missing dates, but after being visited to advise me I can see that things are going on well I am keeping the dates, I am not missing the taking medication and I am taking following the date.* **Trace client in NN-participant 09** |
|  |  | ·       *TRACE has helped a lot. In short, it has helped in saving my life by reminding me to go and get my drugs and also sometimes they ask you are ready to go to the hospital and get your medication.* ***Trace*** ***client in NN-participant 25*** |
|  |  | ·       *…I was not looking after my life, when they visited me and told me that they are from the hospital, I was very happy and in knew that I was playing too much and I changed I don’t miss medication even when I am drunk according to what trace team told me, even when I am drunk I take medication in the morning.* ***Trace client in NN-participant 13*** |
|  |  | ·       *…*right now it has impacted me and encouraged me to take medication properly. ***Trace client in NN-participant 13*** |
|  |  | ·       *it was beneficial, it made my life change, the first thing that changed is my mind I don’t want people to visit me again and tell me that I stopped taking medication aaah no, 2, the other thing that changed is that my body has changed it is not the way I felt last time, the only problem that is left and got worried is the feet problem that they produce heat, my health status has changed even the body weight, and sometimes I feel that things are now working.* ***Trace client in NN-participant 15*** |
|  |  | ·        *…for the job they are doing, with the trace program they are doing I can see my life is moving forward and I cannot stop taking medication because they take time to advise me encourage me and I appreciate the advice they gave me is helping me in terms of my life.* ***Trace client in NN-participant 17*** |
|  |  | ·        *I stay I was experiencing some nausea , just wanted to sleep and my feet were swollen, but the moment the people visited me and went to the hospital the legs got back to normal, the complications ended there and I was energized, I was failing to carry a bucket of water but now I can and I can also farm, I can see change the time I stopped taking medication I was not looking like a human I was even carried away by the wind but the moment they came and told me to go the hospital to collect medication and when I started taking medication I was energized and my energy got back to normal.* ***Trace client in NN-participant 18*** |
|  |  | ·       *I saw change because my legs got normal and I was energized, when I was not taking medication my legs got swollen and I when I wanted to work I was failing and when I started taking medication I was energized and everything got to normal.* ***Trace client in NN-participant 18*** |
