# Supplementary material for: Re-engagement and retention in HIV care after preventive default tracking in a cohort of HIV-infected patients in rural Malawi: A mixed-methods study
Source: PLOS Glob Public Health. 2024 Feb 21;4(2):e0002437. doi: 10.1371/journal.pgph.0002437 (PMC10880992; doi:10.1371/journal.pgph.0002437)
Supplement: Attachment — Submitted filename: Reviewer Comments_TRACE.docx [file pgph.0002437.s005.docx]

Thank you for the opportunity to read and review this paper. Overall, I found the paper to be very interesting and relevant. Some sections will quite a lot of revision to improve the manuscript. Please find my comments below:

ABSTRACT

Overall, the abstract and key messages read very well. However, please note the following edit:

- What OR adjusted for? This isn’t clear
- The comma is misplaced between “patients ‘outcomes”… It should read “patients’ outcomes”

BACKGROUND

Very well written. However, please note the following edits:

- You have “by 20203” and should read “ by 2023”
- Once loss to follow up is abbreviated, it should read “LTFU” throughout the manuscript
- More detail is needed in describing the TRACE programme here. I can see that more detail is provided in methods section but I think a more detail is needed or atleast referenced here

METHODS

- Should read “via a paved road” not “by a paved road”
- Missing references for APZU information including the IC3 model
- How were participants purposively chosen for the interviews? By age/gender?? This is missing here
- Given that you participants younger than 18 years in the study, why was ethics not sought to facilitate consent/assent for them? Would have really interesting to hear their perspectives as well

RESULTS

- There are two Table 3s
- Quote on page 27 doesn’t really support the “caring approach” described. I would replace with more appropriate quote
- I am not sure I see nor understand the difference between “re-shaping mindset” and “motivation” here. Perhaps you could make this more clear.

DISCUSSION

I would say that this section needs the most revision. Please see suggested edits and comments below:

- I don’t think you can say TRACE is reason for re-engagement… It may be one the reasons as your results show but there could be other reasons as well e.g. other existing programmes in the area
- “At 6, 12, and 24 months, respectively, 88%, 84%, and 82% of patients traced were still alive and active on ART at the time of this analysis. Our study sheds light on RiC post-tracking at three points in time, representing a unique aspect of this study given that in most studies, only characteristics, facilitators, and barriers to re-engagement have been explored (53).” – Not sure what this adds to the discussion. I would remove.
- “Furthermore, a study conducted in Latin America showed that patients with WHO stage IV presented a high risk of dying in care even after ten years, regardless of events between enrollment and death (55). Despite this finding, a study on frequent disengagement and re-engagement in Kenya showed that patients at WHO stage IV were less likely to return to care after tracking. In the Sub-Saharan region, ART patients at WHO stages III and IV need more support to reach the ART care facilities (57). ” – This paragraph structure doesn’t make sense either. Paragraph should start with topic sentence and then supported by evidence in following sentences. Should be revised.
- “Second, initiating ART during…” – second to what?
- These findings call for gender-focused approaches, such as in male clinics, when addressing retention and re-engagement in care.” – I don’t find this sentence very strong at all… I would revise to say something about how your findings, unlike others, suggest that women also need to be focused on in interventions.
- “the needs of specific groups” – give examples based on your results
- “we did not explore other factors that could significantly influence patients’ re-engagement in HIV care, such as distance from health facilities, socioeconomic status, transportation, and geography” – why wasn’t this done?
