# Supplementary material for: Re-engagement and retention in HIV care after preventive default tracking in a cohort of HIV-infected patients in rural Malawi: A mixed-methods study
Source: PLOS Glob Public Health. 2024 Feb 21;4(2):e0002437. doi: 10.1371/journal.pgph.0002437 (PMC10880992; doi:10.1371/journal.pgph.0002437)
Supplement: Attachment — Submitted filename: Response to Reviewerss.docx [file pgph.0002437.s006.docx]

**Response to Reviewers**

- **We appreciate the reviewer’ s positive view of the overall value of the paper.**

Reviewer #1: Thank you for the opportunity to read and review this paper. Overall, I found the paper to be very interesting and relevant. Some sections will quite a lot of revision to improve the manuscript. Please find my comments below:


ABSTRACT
Overall, the abstract and key messages read very well. However, please note the following edit:
- What OR adjusted for? This isn’t clear

- **Thank you to the reviewer for this question. We adjusted for gender, BMI, age, and the timing of ART initiation. ( Line 41). We thank the reviewer for this remark. We have added “odd ratio” before “OR’ to clarify what aOR stands for. (Line 43)**. **We have added “confidence interval” before “CI” to clarify what “CI” stands for. (Line 43)**

- The comma is misplaced between “patients ‘outcomes”… It should read “patients’ outcomes”

- **We thank the reviewer for the correction. We corrected the misplaced apostrophe, now read “patients’ outcomes”(Line 43)**

BACKGROUND
Very well written. However, please note the following edits:

- You have “by 20203” and should read “ by 2023”

- **We thank the reviewer for this remark. We have corrected the year now it read “2030” as recommended.**

- Once loss to follow up is abbreviated, it should read “LTFU” throughout the manuscript.

- **We thank the reviewer for this remark. We have abbreviated loss to follow up throughout the entire manuscript as recommended.**

- More detail is needed in describing the TRACE programme here. I can see that more detail is provided in methods section but I think a more detail is needed or at least referenced here

- **We thank the reviewer for the comment on TRACE description and we have added more information about TRACE program in paragraph 5 of the introduction section. ( Line 170 and 171), The description of the program is done in the method section. (Line 274-302)**

METHODS
- Should read “via a paved road” not “by a paved road”

- **We have corrected as recommended now read “via a paved road” (Line 222)**

- Missing references for APZU information including the IC3 model

- **We have added the missing reference as recommended. (Line 231)**

- How were participants purposively chosen for the interviews? By age/gender?? This is missing here.

- **Participants were purposefully selected by health center location. (2 participants per facility) (Line 218)**

- Given that you participants younger than 18 years in the study, why was ethics not sought to facilitate consent/assent for them? Would have really interesting to hear their perspectives as well

- **Thank you for this remark on younger participants. We used secondary data but for the qualitative arm we selected participants above 18 years only. The perspectives of the younger participants would have been indeed interesting, but the team decided to limit the scope of the study to adults (18 year+ and individual that are able to provide consent). We believe that this group will be the focus of future studies in Neno.**

RESULTS

- There are two Table 3s

- **We thank the reviewer for this remark. Tables re-numbered properly now as recommended. Table 4 reference in line 332, Table 5 in line 448, Table 6 in line 465, and Table 7 in line 476.**

- Quote on page 27 doesn’t really support the “caring approach” described. I would replace with more appropriate quote	-

- **We thank the reviewer for this remark. The “Caring approach” sub section is supported by 3 quotes from participants #18 in lines 545 to 550, participant #10 from line 554 to 556 and participant #11 from line 561 and 562. We took your remark into deer consideration but quotes from participant #18 is telling a story about how his stigma at home made him stop treatment and seeing TRACE trackers following him like friends who care convinced him to focus on his treatment instead of the stigma. We were unable to find another one that could express the “caring approach” of the TRACE trackers.**

- I am not sure I see nor understand the difference between “re-shaping mindset” and “motivation” here. Perhaps you could make this more clear.

- **We thank the reviewer for this remark on the difference between “re-shaping mindset” and “motivation”. Since the headers for subsection 3 and 4 in section qualitative results seem to be similar, we have reframed and re-titled section 3 as “Motivation to resume care” and section 4 as “Ownership of care and of personal responsibility”.**

DISCUSSION
I would say that this section needs the most revision. Please see suggested edits and comments below:
- I don’t think you can say TRACE is reason for re-engagement… It may be one the reasons as your results show but there could be other reasons as well e.g. other existing programmes in the area

- **We reframed the first part of the discussion to show TRACE program was associated with high rate of re-engagement instead of been the one reason for the results shown which now presents TRACE as a contributor among other existing programs activities etc… that may influence patients to return to care. This paragraph now read ( Lines 658-667):**

*” We observed that TRACE was associated with high rate of re-engagement, 96% of HIV clients with missed appointments returned to care after being a TRACE visit. This high re-engagement rate shows how a community outreach strategy such as TRACE, even in rural and hard-to-reach areas in low- and middle-income countries, can contribute to address the adverse effects of LTFU and defaulting from care. Our results concur with similar tracking programs implemented in Sub-Saharan Africa, demonstrating the positive impact and high re-engagement rate of clients who disengaged from HIV care (51,52). Zhou et al., when analysing data from the Asia-Pacific region, predicted that the risk of being permanently LTFU increases with the number of days out of care (53). As such, TRACE helps mitigate the negative effects of LTFU.”*

- “At 6, 12, and 24 months, respectively, 88%, 84%, and 82% of patients traced were still alive and active on ART at the time of this analysis. Our study sheds light on RiC post-tracking at three points in time, representing a unique aspect of this study given that in most studies, only characteristics, facilitators, and barriers to re-engagement have been explored (53).” – Not sure what this adds to the discussion. I would remove.

- **Thank you for the reviewer for the remark on this paragraph. This section was removed as recommended.**

- “Furthermore, a study conducted in Latin America showed that patients with WHO stage IV presented a high risk of dying in care even after ten years, regardless of events between enrollment and death (55). Despite this finding, a study on frequent disengagement and re-engagement in Kenya showed that patients at WHO stage IV were less likely to return to care after tracking. In the Sub-Saharan region, ART patients at WHO stages III and IV need more support to reach the ART care facilities (57). ” – This paragraph structure doesn’t make sense either. Paragraph should start with topic sentence and then supported by evidence in following sentences. Should be revised.

- **We thank the reviewer for this remark. We reworked this section now read: (Line 669-702)**

*Patients presenting as WHO stage IV during their ART enrollment were more likely (than stages I-III) to be active in care after 6- and 12 months post-tracking. The literature on retention post-tracking is scarce; therefore, the significance of this finding might be specific to patients in the Neno District. In other areas, a high attrition rate has been reported for patients enrolled at WHO stage IV. For example, Mutasa-Apollon et al. found in a study in Zimbabwe that clinical stage IV was one of the factors associated with a high attrition rate (71). Furthermore, a study conducted in Latin America showed that patients with WHO stage IV presented a high risk of dying in care even after ten years, regardless of events between enrollment and death (72). Despite this finding, a study on frequent disengagement and re-engagement in Kenya showed that patients at WHO stage IV were less likely to return to care after tracking (73). In the Sub-Saharan region, ART patients at WHO stages III and IV need more support to reach the ART care facilities (74).*

- “Second, initiating ART during…” – second to what?

- **We removed “Second “to avoid any confusion. (Line 704)**

- These findings call for gender-focused approaches, such as in male clinics, when addressing retention and re-engagement in care.” – I don’t find this sentence very strong at all… I would revise to say something about how your findings, unlike others, suggest that women also need to be focused on in interventions.

- **We Thank the reviewer for pointing that out, the statement** *"These findings call for gender-focused approaches, such as in male clinics, when addressing retention and re-engagement in care.”* **was removed. Now read**, *" These findings suggest that retention in care programs need to focus on women need.”* **To make the sentence stronger.**

- “the needs of specific groups” – give examples based on your results

- **Thank you to the reviewer for asking, examples are given, now the sentence reads** **(Lines 743 and 744)** *" Our results suggest that, when designing programs to mitigate LTFU, the needs of specific groups such as older patients (30 year old and plus) must be taken into account to address these needs efficiently."*

- “we did not explore other factors that could significantly influence patients’ re-engagement in HIV care, such as distance from health facilities, socioeconomic status, transportation, and geography” – why wasn’t this done?

- **We thank the reviewer for this question regarding other factors that could influence re-engagement. While, distance from health facility, socioeconomic status and transportation are indeed crucial to understanding patient retention, the goal of our study was to explore the experiences of TRACE recipients and to assess the role of TRACE in re-engagement in care. Therefore, the study was designed to focus on TRACE practices and experiences and not on other factors impacting retention"**

Reviewer #2: This paper reports on an intervention (TRACE) designed to reduce loss-to-follow-up (LTFU) among patients attending the IC3 (integrated chronic conditions services) in Neno District, Malawi. LTFU is indeed a serious barrier to achieving HIV treatment goals (and presumably those for other diseases as well). The study presents results from a mixed method evaluation of TRACE as conducted over 2 years, but without any comparison site/ counterfactual. As such, it would benefit from being re-written as a process evaluation that examines some of the challenges, successes, opportunities and - crucially - mechanisms of action for how the intervention was implemented and experienced. At the moment, the paper remains at a fairly descriptive level and lacks conceptual and contextual data that would help readers understand the intervention better, and how it was theorised to reduce LTFU and whether these hypothesised pathways of change were achieved in reality. More specific feedback for each section is provided below.

INTRODUCTION - the authors are correct to identify LTFU as a significant challenge, and one that continues to undermine HIV treatment success in Malawi and elsewhere. However, despite referring to literature that has examined some of the reasons behind LTFU (eg ref #7) these reasons are not presented, nor how different models for improving retention in care/ reducing LTFU are theorised to address these reasons (text messages, peer counsellors, patient navigators etc). These interventions were presumably designed with different theories of change in mind, i.e. how they might address barriers at different levels such as psychosocial / individual motivation, fear of stigma/ disclosure, lack of transportation or travel distance, etc. This kind of background information would be relevant to presenting TRACE and its guiding principles so readers understand its specific rationale.

- **We thank the reviewer for these remarks. We took them into consideration and reasons behind LTFU and presentation of different models to address LTFU are now added to paragraph 3 (new paragraph) of the Introduction section (Lines 120-153). TRACE guiding principle are presented in paragraph 5 of introduction section (Lines 170-175). and in sub-section TRACE Strategy (Lines 275-302)**

METHODS - Related to the above, more detail about the rationale for TRACE (and its "active ingredients") is required. Why were CHW selected rather than other forms of patient-centred support?

- **We thank the reviewer for this question, an additional sentence was added to explain why CHW were used. (Lines 283-284)**

*“TRACE program relies on CHW because of their complete integration in the communities, health facilities and district health programs.”*

What does patient-centred support actually include in the context of the IC3 programme?

- **We thank the reviewer for this question on how the IC3 programme and its patient centered attribute. The concept of patient centered approach transpired in IC3 programme through different aspects describes in the article.**

**1) The core concept of IC3 is increase the proximity to patients, by decentralizing the care to bring the services closer to patients IC3 improves access (Line 250).**

**2) Patients during their visit are allocated time for group and individual counselling with their providers or lay counsellors and to improve patient experience. (Line 257-258)**

**3) Confidentially is also core and can be seen in the way patient chart are handled at IC3 which reduces stigma. (Line 256-257)**

**4) All services at IC3 are free of charge to increase assess since most patients in Neno are living below the poverty line. (Line 250-251)**

**5) One-stop-shop characteristic of the IC3 concept is important element of patient centered care, Patients are seen holistically during IC3 visit. (Line 256)**

What were CHW tasked with doing, and which potential determinants of LTFU were they targeting specifically?

- **We thank the reviewer for this question. The tasks attributed to CHWs are described in the text. (See sub-section Neno Model of care) (Line 241-249)**

*” CHWs work completement and alleviate the burden on Health Surveillance Assistant, ministry ‘s of health cadres that are often overworked within the health system in Malawi(58,59). Each CHW regularly visits 20 to 30 households monthly and provides an initial screening assessment for conditions such as HIV, malnutrition, maternal and child health, mental health, noncommunicable diseases (NCDs), basic hygiene, and health education (60). Depending on the outcome of the screenings, household members are referred to health facilities for diagnostics and management. When CHWs visit the households, they provide treatment support to patients already enrolled in various care programs at the facilities. “*

…which potential determinants of LTFU were they targeting specifically?

- **We thank the reviewer for this question on determinant of LTFU targeted by CHWs. In relation to the potential determinant of LTFU, aided by EMR lists CHWs locate patient with missed appointment, visit them, they provide early reminder counsel and encourage patients to contact the clinic services for psycho-social, economic, medical, and environmental reasons for the missed appointment. (See sub-section TRACE strategy). (Line 281-291)**

*“Twice a month a tracking list of patients who have missed appointments for 2, 4, or 6 weeks or more is generated using EMR. The tracking list is sent to the CHW team to ensure the tracking of missed appointments of under 6 weeks, whereas the TRACE trackers track patients who have missed appointments for six weeks or longer. TRACE rely on CHW because of their complete integrations within both communities and health facilities and district health programs. During the visit, the CHWs remind patients of their missed appointment and inform them to return to the facility for a visit, which will be updated in the EMR system. In addition, CHWs collect information and refer (sometime accompany) patients to the clinic for linkage to medical and psycho-social services. Therefore, patients on the TRACE trackers list already had 2 visits from the CHW team which constitute early reminder.”*

There is very little detail here - are CHW volunteers or a paid cadre within the health system?

- **Details about CHW status added in Subsection Neno Model of Care. (Line 240-241)***.*

*“CHWs are receive training, ongoing education, service delivery tools and a monthly volunteer stipend (38USD).”*

Why were "trackers" added after 6 weeks following missed appointment?

- **We thank the reviewer for this question on why TRACE trackers were added after 6 months of missing appointment. We re-worked the sentences about TRACE tracker task and rationale in the manuscript for more clarity to the readers. We explained that TRACE trackers are used for deeper psycho-social counselling to patients. Their work goes beyond the simple reminder but assess and counsel patients and refer to the clinic for psychosocial and economic or nutrition assistance to patients. As explained in sub-section TRACE strategy. (Line 292-299)**

*”” In that case, the TRACE tracker is now sent to the patients’ homes to remind them of their appointment, assess why they missed the appointment, and refer patients to appropriate programs for follow-up like psycho-social, economic assistance service and nutrition services. During this third visit the patient receive a deeper psycho-social counselling by TRACE tracker.”*

What do trackers do that CHW do not? What was the theorised pathway from adding the TRACE tracking to improved retention in care? (was it simply about reminding and motivating patients, or was there any kind of psychosocial mechanisms such as offering social support, increasing problem-solving skills, etc?)

- **We thank the reviewer for these questions. We added more details to help clarify the readers. Trackers provide deeper counselling to patient with missed appointments. As explained in the sub-section TRACE strategy. (Line 292-299)**

*“ In that case, the TRACE tracker is now sent to the patients’ homes to remind them of their appointment, assess why they missed the appointment, and refer patients to appropriate programs for follow-up like psycho-social, economic assistance service and nutrition services. During this third visit the patient receive a deeper psycho-social counselling by TRACE tracker.”*

Qualitative methods - how were interviewees purposively selected (ie based on what criteria and why?)

- **We thank the reviewer for this question on selection of participants for the study. Selection was based on willingness to participate, and ability to provide consent. More details are added in sub-section Qualitative data collection. This sub-section now read:(Line 383-392)**

*“From the pool of participants, 25 were interviewed instead of the 26 who had been scheduled for interviews, as one had withdrawn from the study after being contacted. Recruitment and interviews were conducted between August to December 2021. Twenty-five in-depth semi-structured interviews were conducted with ART active patients purposefully selected from the list of patients tracked preventively between January 2018 and June 2019. Selection was based on good retention after successful tracking by TRACE team and willingness to participate in the study. Only patients 18 years of age or older, who could give consent, were included in this study. The interviews were conducted in the Chichewa language, recorded, and transcribed. The interviews aimed to capture the tracking elements from the patients’ perspectives and the impact of the tracking on patient retention and re-engagement in care.”*

Quantitative methods - why were the specific variables selected for comparison? Age and sex are fairly obvious (I think the researchers asked about sex rather than gender?) but why BMI and TB status?

- **We thank the reviewer for this question. The selection of specific variables aimed at presenting a complete description of the participants characteristics.**

RESULTS: For background context, how many (and what %) of individuals were NOT reached by the CHW or TRACE trackers, and why not? (not home, refused entry into the home). As the paper is focused just on those individuals who were successfully identified by TRACE trackers, it's hard to get a perspective on trackers' overall ability to find the identified individuals. We know 1100 were visited at home, how many did trackers try and fail to visit?

- **We thank the reviewer for this question on percentage of patients with missed appointment on TRACE lists not tracked by TRACE. According to the TRACE design all patients with a missing appointment are listed to be visited. A patient is considered traced whenever the tracker reaches out to the patient’s location regardless of the outcome of the tracking (patient found, patient not found, patient moved out, patient hospitalized, patient dead). Tracing intervention for patients who missed appointments or refill for 6 weeks or more yielded a 100% contact success rate for the period and through the contacts we were able to determine various clinical outcomes like default, transfer out, died and alive on ARV treatment. However, some patients might not be traced the exact scheduled date for technical reasons like incapacity to reach patients during rainy seasons, during cyclonic etc, but are usually traced as soon as the technical situation is amended. No failure to tracked have been reported for the study period.**

I found the division of findings across the 3 time periods very confusing, and not something that was addressed in Discussion. Perhaps there is a way to synthesise the time periods by describing temporal trend? For example, stressing that while certain types of patients are more likely to be retained over the shorter term, other categories of people may experience longer-term barriers.

- **We thank the reviewer for this question and suggestion but retention in care generally is measure at some points in time like 6 months retention, 12 months retention, 24 months retention ect…Retention at different point has been used at program performance indicator widely. Reporting the proportion of patient retained at 6, 12, 24 months (as well as associated factors) post tracking was a way to show the positive impact of TRACE on patient retention.**

Also, do the socio-demographic profiles of those tracked by TRACE differ substantially from those who never missed appointments (or were missing for fewer than 6 weeks and thus were never eligible for TRACE)?

- **We thank the reviewer for this question on patients who never missed appointments and comparison group. We did not build a comparison group of patients who never missed appointments, but we can assume that the demography in rural Neno is quite homogenous, if a difference exist it might not be significant.**

Page 23 - in the paragraph directly preceding Table 6 there is a contradiction regarding whether or not age is associated with RiC at 24 months. Age and BMI are stated as not being associated but an earlier sentence includes "older people" as a category with lower RiC (compared to being male and starting ART post-2016).

- **Please see paragraph preceding table 7 entitled “Factors associated with 24-month return to care and mortality outcomes among HIV-infected male patients on antiretroviral therapy (ART) “ the last sentence reads as follow: (Line 484)**

*“Age and BMI were not associated with RiC at 24 months.”*

Qualitative results - these offer the possibility of examining "mechanisms of action" of the TRACE intervention, but again, the lack of any context regarding how the programme was designed, the activities of the trackers and how these were theorised to change behaviour, make it difficult to know how the researchers identified their themes. The qualitative analysis reads like formative research, the kind of work that should have been conducted before designing an intervention in order to identify relevant barriers to RiC that the trackers could target. Given the focus on "motivation" and "reshaping mindset" in the results, I am assuming the researchers wanted the intervention to focus on individual-level determinants of LTFU. What about addressing some of the broader issues like transportation difficulties, fear of stigma and disclosure, isolation and poor social support etc? Is there a reason that these were not part of the trackers' focus and did any of these emerge from the data?

- **We Thank the reviewer for the comment and question. The issue of framing the goal was targeting understanding why patients came back instead of what make them miss appointment.**

I wonder if findings could be re-structured to start with the qualitative results and link these to how the programme was supposed to create behaviour change, and then identify for which kinds of people (and through what processes) this appeared to happen (or happen more or less). Again, the structure and concepts from process evaluation (including any challenges of implementation) might be a useful framework for this paper.

- **We thank the reviewer for the suggestion, to which we gave deer consideration but from a design perspective, the qualitative findings are to explain the quantitative results (explanatory sequential design) therefore alternating how the result section is presented will change the design all together.**

DISCUSSION - the researchers highlight where there findings differ from that in the literature (e.g. younger and male patients displayed better RiC following tracking). Is there any qualitative data that helped explain this? The two data sets could have been better combined in this section, to give a holistic picture of what the barriers might be for the different socio-demographic groups and how the work of the trackers appeared to reduce these.

- **We thank the reviewer for this comment and question about qualitative data explaining result on younger and male displaying better RiC but we did not find anything in the qualitative data to explain this particular finding.**

OVERALL: It is laudable that the intervention was able to maintain high rates of RiC / prevent further LTFU for patients who were visited by the TRACE programme, however, we still don't know whether this was a large proportion of those it aimed to contact in the first place, and there is little information for other programmes in terms of how the programme works, for whom, and in what circumstances so that the lessons learned can be feasibly replicated elsewhere.

- **We thank the reviewer for the overall comment on this manuscript.**
