# Supplementary material for: Re-engagement and retention in HIV care after preventive default tracking in a cohort of HIV-infected patients in rural Malawi: A mixed-methods study
Source: PLOS Glob Public Health. 2024 Feb 21;4(2):e0002437. doi: 10.1371/journal.pgph.0002437 (PMC10880992; doi:10.1371/journal.pgph.0002437)
Supplement: Attachment — Submitted filename: Review Manuscript Number PGPH-D-23-01074R1.docx [file pgph.0002437.s007.docx]

**PLOS Global Public Health**

**Re-engagement and retention in HIV care after preventive default tracking in a cohort of HIV-infected patients in rural Malawi: A mixed-methods study**

**Manuscript Number: PGPH-D-23-01074R1**

**Reviewer Report**

| **Manuscript text** | **Comment** |
| --- | --- |
| LINE NUMBERS | Next time please include line numbers as it makes it easier for the reviewer and you in correcting. |
| *A back-to-care program was implemented in urban Lilongwe, Malawi; 85% were successfully traced, and reasons for patients defaulting from the HIV-care program were explored (37).* | Check sentence not sure there should be a ; there. Also, maybe briefly state the reasons for patients defaulting. |
| *The integrated chronic care platform is designed as a hub-and-spoke model, where the two hospitals provide additional staff, supplies, and medications to support the primary health center “spokes,” which are visited weekly or fortnightly for IC3 clinics.* | What are spokes? |
| *From the pool of participants, 25 were interviewed instead of the 26 who had been scheduled for interviews, as one had withdrawn from the study after being contacted.* | Please state reason for withdrawal if known. |
| *Twenty-five in-depth semistructured interviews were conducted with ART active patients purposefully selected from the list of patients tracked preventively between January 2018 and June 2019.* | Define purposefully selected and provide a citation. |
| *The coded data were summarized and analyzed using content analysis for emerging themes and categories discussed by the study team.* | Define content analysis and provide a citation. |
| *All participants interviewed in the qualitative phase of this study gave written consent or oral consent if they could not read, in which case the consent was read to them, and oral consent was given.* | I assume it was because they could neither read nor write in English. Please be clear. |
| ***Participant #17, woman, 43 years old.*** | Sometimes it is nice to give an additional indicator about the participant as it relates to the research question, for example, education or occupation. |
| ***Participant #25, man, 43 years old***  ***" Participant #13, woman, 56 years old.*** | Please move these lines down below the quotations to maintain the correct formatting. |
| NUMBER AND FORMAT OF QUOTATIONS | Just a suggestion but you could instead write one paragraph of summarizing text and then put the relevant quotes in a table format to streamline as the quotes are making the results section longer. |
| *With this intervention, 96% of HIV clients with missed appointments returned to care.* | I like that your discussion section starts by pointing out the strength of the study |
| *Furthermore, a study conducted in Latin America showed that patients with WHO stage IV presented a high risk of dying in care even after ten years, regardless of events between enrollment and death (55).* | It seemed this paragraph should be merged with the one prior. Make sure to keep paragraphs with a similar idea/messaging formatted together. |
| *Second, initiating ART during the test-and-treat era was found to be highly… Third, at 24-month post-tracking, men in this study were more likely to be active in ART…* | None of the previous paragraphs open up with First, … |
| *Nevertheless, tracking that has a clear purpose and is well explained to patients, is informative, and can convey a message of acceptance and friendship – as shown in our study results – is more likely to achieve re-engagement and positive outcomes.* | Re-word this sentence as it is busy and not clearly communicating the intended message. |
| *First, it was conducted in one district with very distinct characteristics.* | Not clear. What is unique about the district that makes it a limitation? |
| *Third, the time between ART enrollment and patient outcome was not factored into our analysis, nor was the time between tracing and re-engagement.* | Why not? |
