# Supplementary material for: Re-engagement and retention in HIV care after preventive default tracking in a cohort of HIV-infected patients in rural Malawi: A mixed-methods study
Source: PLOS Glob Public Health. 2024 Feb 21;4(2):e0002437. doi: 10.1371/journal.pgph.0002437 (PMC10880992; doi:10.1371/journal.pgph.0002437)
Supplement: Attachment — Submitted filename: Response to the reviewers..docx [file pgph.0002437.s008.docx]

**PLOS Global Public Health**

**Re-engagement and retention in HIV care after preventive default tracking in a cohort of HIV-infected patients in rural Malawi: A mixed-methods study**

**Manuscript Number: PGPH-D-23-01074R1**

**Response to Reviewers**

**Responses to reviewer #2**

| **Manuscript text** | **Comment** | **Responses to reviewers (Dimitri)** |
| --- | --- | --- |
| lines 124-125 | Reviewer #2: I think the Introduction needs to be revised to make clear that this is not an evaluation of the TRACE programme (lines 124-125 imply that an evaluation is being reported). It is a descriptive study of the characteristics and experiences of participants over a specific period. It is not an outcome evaluation because there are no comparisons made to previous trends or other sites or populations, and it is not a process evaluation because here are no clearly identified process variables related ti implementation such as fidelity, feasibility, quality etc. | **Thank you very much for this point and agree with your observations. The abstract and introduction was re-worked to align with this recommendation. We made it clear now that the manuscript is presenting a description instead of an evaluation. See line 221 to 226**  “However, the program has not been described and further knowledge of the TRACE program will serve to inform design and implementation of community interventions that aim to prevent LTFU and increase patient re-engagement. Therefore, by using a mixed-methods approach, we aimed to present a description of patient re-engagement, RiC, and patient characteristics after being tracked by the TRACE program along with program acceptability from the perspectives of the beneficiaries.” |
| line 629 | Reviewer #2: In the Discussion, I suggest removing the hypothesised counterfactual on line 629 that including comparison to non-tracked patients would "... would have amplified the effect" - this implies that you are attributing the observed rates of retention in care to the intervention, which you absolutely cannot do with the data presented. There is an association, but you have no way of being sure that there were not exogenous factors at play (a coincidental media campaign, a different health intervention, a change in public health facility protocols or even just a random change in health seeking behaviour). | **We thank the reviewer for this recommendation. The hypothesized countefactual on line 629 was removed as recommended by the reviewer. Now read:**  ” Fourth, a comparison group was not included in the analysis as all patients who missed appointments in the district were traced according to TRACE protocol” (line 654-657) |
|  | Given that the paper is descriptive - and, indeed, useful as an indicator of the type of proactive preventive approaches that can be taken with LTFU - I would still like to see a bit more detail on the way that the intervention approached determinents of LTFU. | • **We thank the reviewer for this remark. TRACE program identifies reasons for missing appointment and refer to appropriate program within APZU for intervention. More details provided (217-223). The last part of section of TRACE strategy” now read:**  “The length of this visit varies from few minutes to half an hour accordingly to nature of the reason for missing schedule appointments. After the tracker introduce her/himself, states the purpose of the visit, time is given to patients to share their concerns and/or challenges and to discuss possible solutions with the tracer. At the end patients are referred to appropriate APZU programs like clinical programs, psycho-social, financial assistance service, and nutrition services to address possible root causes for missing appointments. Reference to appropriate services is done first verbally referral with patient in addition to electronic referral (with patient’s details) is sent to the receiving programs.” |
| line 181 | The paper mentions being clear in purpose and "person centred" but no details are given in terms of what the TRACE workers did when they say someone, how long a visit or call was normal, what kind of counselling or problem-solving was offered, the health promotion messages included etc. Was there a manual or guidelines to follow, or were TRACE staff trained in differentiated support? There is little here that someone keen to replicate the programme in a similar context would be able to adapt and try. This level of detail would also help contextualise the qualitative data on acceptability, so that readers could understand which components were most appreciated by LTFU patients, and what likely mechanisms of action for successful RiC were. I suggest expanding the TRACE Strategy section that starts on line 181. | • **We thank the reviewer for these comments and questions. TRACE is person-centered intervention in way that it allows patients to share their concerns and challenges during the tracing. Patients are involved in the brainstorming for solutions to missed appointments. More information are added to the TRACE strategy sectio**n" (Line 208-219) |

**Reviewer #3**

| LINES | Next time please include line numbers as it makes it easier for the reviewer and you in correcting. | **We thank the reviewer for this suggestion, numbers have been added to each line for easy review.** |
| --- | --- | --- |
| *A back-to-care program was implemented in urban Lilongwe, Malawi; 85% were successfully traced, and reasons for patients defaulting from the HIV-care program were explored (37).* | Check sentence not sure there should be a ; there. Also, maybe briefly state the reasons for patients defaulting. | **We thank the reviewer for this remark and suggestion, which we accept gladly. We removed the sentence, and paragraph 3 of the introduction was re-worked for more clarity. (Line 82 to 101)**  “In Sub-Saharan Africa reasons for LTFU range from individual (psychological) to socio-economic and programmatic. The current literature describes two levels of barrier to retention leading to LTFU. A first level of barriers that are more socio-economic programmatic and environmental such as financial difficulties (transportation costs, lack of food, inability to pay service fees), mismatched scheduling, unexpected events, distances to health facilities, which are found to be associated with high rate of return to care (20–24). A second level of barriers are more medical and psychological such as side effects, forgetfulness, fear of stigma, lack of motivation, denial, personal beliefs, treatment fatigue, which requires focused inter-personal interventions (21,25,26). To address these barriers, the World Health Organization (WHO) has recommended that HIV care programs implement clinical and community-based interventions to support ART adherence and patient RiC (27,28). The current body of literature reports that interventions targeting both level of barriers, have been proven cost-effective in preventing LTFU (29–33). Programmatic interventions like transport reimbursements, direct cash transfer, newer drug formulation, differentiated health care service delivery have been implemented in Sub-Saharan Africa to address socio-economic and medical causes of LTFU (34,35). In addition, interpersonal intervention examples for LTFU patients including phone calls, text messages, peer counsellors, patient navigators, or lay counsellors to trace LTFU patients were proven to be successful interventions in addressing medical and psychological barriers to retention (30,32,34,36–41). |
| *The integrated chronic care platform is designed as a hub-and-spoke model, where the two hospitals provide additional staff, supplies, and medications to support the primary health center “spokes,” which are visited weekly or fortnightly for IC3 clinics.* | What are spokes? | **We thank the reviewer for this question. The sentence was revised based on the questions and more information is added to clearly present what the hubs and hobs are in our description of “Neno model of care”. Lines 159-163 now read:**  “The integrated chronic care platform is designed as a hub-and-spoke model, where the “hubs” are the two hospitals in the district which exist as the referral centers for chronic care patients. These “hubs” can provide additional staff, supplies, and medications as needed to support the primary health center’s IC3 clinics – the “spokes” in provision of care weekly or fortnightly depending on the number of patients. “ (line 159-163) |
| *From the pool of participants, 25 were interviewed instead of the 26 who had been scheduled for interviews, as one had withdrawn from the study after being contacted.* | Please state reason for withdrawal if known. | **We thank the reviewer for this recommendation. The participant who redrawn did not give the reason for refusing the interview and this has been added to the manuscript. Now lines 293 to 295 read:**  ” From the pool of participants, 25 were interviewed instead of the 26 who had been scheduled for interviews, as one had withdrawn from the study after being contacted without providing an explanation.” |
| *Twenty-five in-depth semistructured interviews were conducted with ART active patients purposefully selected from the list of patients tracked preventively between January 2018 and June 2019.* | Define purposefully selected and provide a citation. | **We thank the reviewer for this question on and suggestion provided to us about the definition of purposefully”. We gladly accepted the suggestion for definition and citations, therefore we added "Selection made for their specifics characteristics (facility, 3 or more tracking visits, re-engaged with care after tracing)" in the text for more clarity. As per the reviewer recommendation we added a citation at the end of the sentence. Now lines 296 to 299 read:**  “Twenty-five in-depth semi-structured interviews were conducted with ART active patients purposefully selected (facility, at least 3 tracking visits, re-engaged in care after tracing) from the list of patients tracked preventively between January 2018 and June 2019 (63,66). |
| *The coded data were summarized and analyzed using content analysis for emerging themes and categories discussed by the study team.* | Define content analysis and provide a citation. | **We thank the reviewer for this question; therefore, we added the definition of " content analysis" as well as appropriate citations. Lines 308-310 now read:**  “The coded data were summarized and analyzed using content analysis, a count-based approach to determine the frequency of themes and categories in a qualitative data set (63,67). |
| *All participants interviewed in the qualitative phase of this study gave written consent or oral consent if they could not read, in which case the consent was read to them, and oral consent was given.* | I assume it was because they could neither read nor write in English. Please be clear. | **We are grateful to the reviewer for this remark. The consent process was done in the local Chichewa language he interviews were conducted in the local Chichewa language to avoid misunderstanding. " Chichewa" is added to the sentence to bring more clarity. Now line 316 to 318 read:**  “All participants interviewed in the qualitative phase of this study gave written consent or oral consent if they could not read, in which case the Chichewa version of consent was read to them, and oral consent was given.” |
| ***Participant #17, woman, 43 years old.*** | Sometimes it is nice to give an additional indicator about the participant as it relates to the research question, for example, education or occupation. | **We thank the reviewer for this suggestion. Indicators about participants such as education and occupation are important to add just as you suggested but the indicators given here are those collected for their relevance to the research question. We thank the reviewer for that as we will surely consider this suggestion in any future work.** |
| ***Participant #25, man, 43 years old*** | Please move these lines down below the quotations to maintain the correct formatting. | **We thank the reviewer for this recommendation to move line down below the quotation to maintain the correct formatting. These lines were moved down as recommended** |
| ***" Participant #13, woman, 56 years old.*** |  |  |
| NUMBER AND FORMAT OF QUOTATIONS | Just a suggestion but you could instead write one paragraph of summarizing text and then put the relevant quotes in a table format to streamline as the quotes are making the results section longer. | **The authors thank the reviewer for this suggestion. the authors would like to maintain the current presentation.** |
| *With this intervention, 96% of HIV clients with missed appointments returned to care.* | I like that your discussion section starts by pointing out the strength of the study | **Thank you, the authors appreciate your encouraging comment.** |
| *Furthermore, a study conducted in Latin America showed that patients with WHO stage IV presented a high risk of dying in care even after ten years, regardless of events between enrollment and death (55).* | It seemed this paragraph should be merged with the one prior. Make sure to keep paragraphs with a similar idea/messaging formatted together. | **We thank the reviewer for this remark, and we accept the reviewer suggestion for the paragraph to be merged with the paragraph 2. Therefore paragraph 2 of the discussion section was reworked. The paragraph was fixed as recommended by the reviewer.** |
| *Second, initiating ART during the test-and-treat era was found to be highly… Third, at 24-month post-tracking, men in this study were more likely to be active in ART…* | None of the previous paragraphs open up with First, … | **We thank the reviewer for this comment and suggestion therefore, we remove “Second” as recommended. The sentence now read as follow: (lines 584_586)**  “Initiating ART during the test-and-treat era was found to be highly associated with being alive and active in ART at 6-, 12-, and 24-months post-tracking when compared to patients who initiated ART before the test-and-treat era.” |
| *Nevertheless, tracking that has a clear purpose and is well explained to patients, is informative, and can convey a message of acceptance and friendship – as shown in our study results – is more likely to achieve re-engagement and positive outcomes.* | Re-word this sentence as it is busy and not clearly communicating the intended message. | **The authors thank the reviewer for this suggestion. This sentence was re-worked for more clarity. Now read**  **“**Nevertheless, tracking that has a clear purpose, well explained to patients, informative, and that can convey a message of acceptance and friendship – as shown in our study results – is more likely to achieve re-engagement and positive outcomes.” |
| *First, it was conducted in one district with very distinct characteristics.* | Not clear. What is unique about the district that makes it a limitation? | **We thank the reviewer for this question. Neno district is unique in Malawi for its remoteness and structure of the health care system supported by PIH. Neno is the only district with this model of care ( IC3 clinic) . Other districts may have NCD clinic but not to this level. As explained in line 648-650 of the manuscript.**  **“**The current study had several limitations and indicated the need for further studies. First, it was conducted in one district implementing a combined NCD and HIV clinic which is not common within the other districts in Malawi where there are separate clinics for these conditions” |
| *Third, the time between ART enrollment and patient outcome was not factored into our analysis, nor was the time between tracing and re-engagement.* | Why not? | **We thank the reviewer for this question about time between ART enrollement. Time between ART enrollment and patient outcome was not of the scope and objectives of this study.** |
